# Benign Vulval Leiomyoma: Report of a Rare Case

**DOI:** 10.7759/cureus.90283

**Published:** 2025-08-17

**Authors:** D. Hassan, S. Percholli Ramasubramanian

**Affiliations:** 1 Obstetrics and Gynaecology, Wythenshawe Hospital, Manchester, GBR

**Keywords:** bartholin cysts, benign tumors, perineal mass, surgical excision of tumour, vulvar leiomyoma

## Abstract

A vulval leiomyoma is an uncommon, benign mass often misdiagnosed as a Bartholin cyst. Its precise etiology is currently unknown, though it typically arises from the deep connective tissue within the labia majora. Clinically, it presents as a slowly developing perineal mass, with pain and distress being the primary symptoms. We present the case of a 41-year-old woman with a one-year history of a gradually expanding vaginal mass. Examination revealed a left-sided vulval mass over 6 cm. Surgical excision was performed, and intraoperatively, an additional 7 cm mass extending deep into the perineum was found on the right vulva, confirming leiomyoma rather than a Bartholin's cyst. Histopathological examination confirmed a smooth muscle tumor with myxoid stroma, no atypia or necrosis, and postoperative MRI showed multifocal confluent soft tissue nodules in the perineum. Differentiation from a Bartholin cyst can be challenging due to similar presenting symptoms, including erythema, irritation, localized enlargement, and an asymptomatic mass. However, specific physical findings, such as inverted labia minora and a firm consistency of the swelling, can support a diagnosis of vulvar leiomyoma, contrasting with the everted labia minora and cystic structure seen in Bartholin cysts. Histopathological examination and MRI findings are crucial for accurate diagnosis and for distinguishing between benign and malignant forms. Surgical excision is the definitive treatment for benign vulval leiomyomas.

## Introduction

Vulval leiomyomas are exceedingly rare benign tumors arising from smooth muscle cells in the vulvar region, accounting for approximately 0.07% of all vulvar tumors [1].

They are often clinically indistinguishable from Bartholin’s cysts due to overlapping features such as localized swelling, erythema, and discomfort. This diagnostic ambiguity often results in delayed or incorrect treatment strategies. While Bartholin’s cysts typically present as soft, fluctuant swellings, leiomyomas tend to be firm, mobile, and may occasionally involve surrounding anatomical structures, thereby altering local tissue morphology [2].

The etiology of vulval leiomyomas remains poorly understood, though they are thought to originate from the erectile tissue, round ligament, or vascular smooth muscle elements in the labia majora. Most patients report slow-growing masses that may remain asymptomatic for extended periods. Nevertheless, larger lesions can produce significant psychological distress and physical discomfort, especially when associated with secondary changes such as cystic degeneration or deep tissue infiltration [3].

In some cases, their growth trajectory mimics that of uterine leiomyomas, although the absence of uterine tissue necessitates histological confirmation [4].

Diagnostic imaging, particularly magnetic resonance imaging (MRI), plays a crucial role in accurately delineating the extent and character of these tumors. However, definitive diagnosis is contingent upon histopathological evaluation, which confirms the presence of smooth muscle fibers without atypia, mitotic figures, or necrosis - hallmarks that distinguish benign from malignant variants [1]. Moreover, MRI can help identify multifocal or recurrent lesions, as in the case presented in this report, where bilateral involvement and perineal extension were observed [5].

This report aims to highlight the diagnostic challenges and management of vulval leiomyomas, using a rare case of bilateral masses that clinically mimicked Bartholin’s cysts. Through detailed clinical, radiological, and pathological evaluation, the case underscores the importance of considering leiomyomas in the differential diagnosis of vulval masses and supports surgical excision as the treatment of choice.

## Case presentation

We present a case of a 41-year-old female patient who had previously undergone a comprehensive laparoscopic total hysterectomy alongside a bilateral salpingo-oophorectomy because of a long history of menorrhagia unresponsive to medical treatment, and who was subsequently started on hormonal replacement therapy. She sought medical consultation at our clinic, reporting a discernible vaginal mass that had been present for a duration of one year, notably exhibiting progressive enlargement over the preceding three months. A thorough local evaluation of the vulva unveiled a substantial left-sided vulval mass, meticulously measured to exceed 6 cm in its maximal dimension.

Subsequent to an exhaustive discussion regarding the diagnostic findings and therapeutic options, the patient provided informed consent and elected to proceed with surgical excision of the mass under general anesthesia. Preoperative assessment, including routine hematological parameters, revealed no significant abnormalities, indicating the patient's systemic health was within normal physiological limits for the scheduled intervention.

During the intraoperative phase, the encountered mass was definitively identified as a leiomyoma, a crucial distinction from the pre-surgical differential diagnosis of a Bartholin's cyst. Furthermore, a careful palpation revealed an additional, previously undetected mass situated on the right vulva, extending profoundly into the perineum, approximating 7 cm in its largest dimension. Subsequent gross pathological evaluation of the excised specimen characterized the tissue as irregular, displaying pale coloration and areas of congestion, with precise dimensions recorded as 12.6 x 5.9 x 2.5 cm. A comprehensive histopathological examination of the resected tissue confirmed the presence of a smooth muscle tumor, notable for its areas of prominent myxoid stroma, and critically, a complete absence of cellular atypia or necrotic foci. This definitive microscopic analysis culminated in a final diagnosis of vulval leiomyoma.

**Figure 1:**
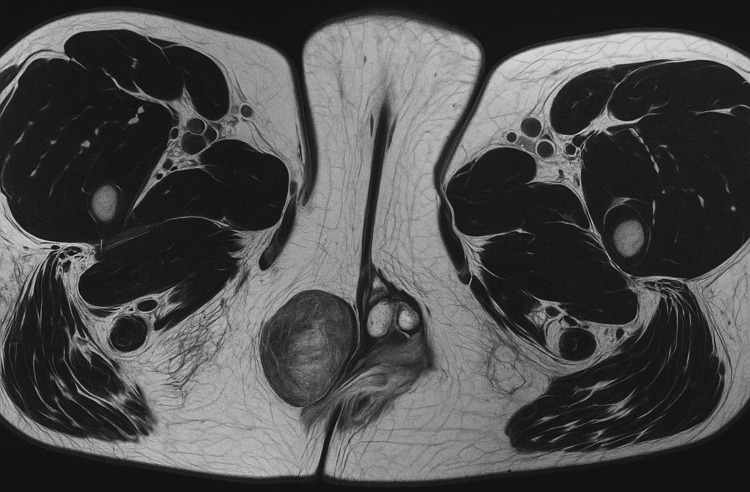
Postoperative MRI of the perineum, demonstrating multifocal confluent soft tissue nodules consistent with vulval leiomyoma.

Postoperative magnetic resonance imaging (MRI) of the perineum, specifically focusing on the right vulva, unequivocally demonstrated the presence of multifocal, confluent soft tissue nodules within the perineal expanse (Figure 1). These nodules were intimately associated with the vaginal introitus and extended posteriorly, partially encircling the anterior sphincter. Notably, several of these identified nodules exhibited an inseparable relationship with the external sphincter. Furthermore, the imaging also revealed the presence of some cystic degeneration within the soft tissue nodule located on the left side.

Clinically, the left vulvar swelling initially mimicked a Bartholin cyst due to its cystic appearance and eversion of the labia minora. However, features such as inversion of the labia minora and the firm consistency of the lesion supported the diagnosis of vulvar leiomyoma. Collectively, the surgical findings, histopathological analysis, and imaging features confirmed the diagnosis of vulvar leiomyoma.

## Discussion

Our case, where a vulval leiomyoma was clinically mistaken for a Bartholin’s cyst, is consistent with previous reports highlighting this diagnostic pitfall. Komatineni and Paul [6] described a 44‑year‑old patient with a 12‑year history of a slow-growing vulval mass that was initially presumed to be a Bartholin cyst until excision revealed leiomyoma.

Similarly, Priya and Lilly [7] reported on a 49-year-old with a painless vulvar swelling, clinically managed as a cyst, but histopathologically confirmed as a leiomyoma.

These cases support the clinical relevance of firm swelling and inverted labia minora as red flags suggestive of leiomyoma rather than a cyst.

The role of imaging, especially transperineal ultrasonography and MRI, is well-documented in distinguishing solid from cystic lesions. Pandey et al. emphasize that transperineal ultrasound can identify the solid nature of leiomyomas, while Weinreb et al. highlight that MRI helps differentiate between benign and malignant variants [8,9].

Studies like Zhao et al. also highlight MRI’s utility in identifying low-signal intensity on T2-weighted images, which may be associated with malignant smooth-muscle tumors but, when not present, further support a benign diagnosis [10]. This aligns closely with our approach using both modalities for more accurate preoperative assessment.

Histopathology remains the definitive diagnostic tool. In a series by Nielsen et al. [11], among 25 vulvar smooth-muscle tumors initially mistaken for cysts, histology confirmed 20 benign leiomyomas, with four atypical and five malignant cases, underscoring the essential need for excision and tissue evaluation. Our patient's benign histology aligns with this typical outcome after complete excision.

Beyond adults, vulvar leiomyomas also affect younger women. A case report of an 18‑year‑old teenager, initially suspected of having a Bartholin cyst, showed how ultrasonography contributed to suspecting a leiomyoma, with histology confirming the diagnosis [12]. This case, together with our report, emphasizes the need for awareness regarding age variations and the importance of imaging and histology in management.

In this case, the persistence and growth of the tumor after bilateral oophorectomy is noteworthy, as leiomyomas typically regress in an estrogen-deprived environment [1]. However, the patient was initiated on hormonal replacement therapy postoperatively, which may have provided sufficient hormonal stimulation to sustain or promote tumor growth.

Treatment and follow-up practices for vulval leiomyomas consistently recommend complete surgical excision with clear margins. Long-term follow-up is encouraged due to the rare but documented risk of recurrence or atypical transformation. Our patient’s benign course post-excision mirrors this standard of care.

## Conclusions

This case highlights the diagnostic challenges posed by vulval leiomyoma, a rare benign tumor often mistaken for more common vulvar lesions such as Bartholin cysts. Our patient, with a history of hysterectomy and bilateral salpingo-oophorectomy, presented with a progressively enlarging vulvar mass that initially mimicked a Bartholin cyst both clinically and on examination. Intraoperative findings, along with detailed histopathological and imaging studies, were essential in establishing the correct diagnosis of vulval leiomyoma and ruling out malignancy.

Accurate identification of vulval leiomyoma relies on a combination of clinical suspicion, surgical exploration, histopathological confirmation, and advanced imaging. Complete surgical excision remains the treatment of choice and offers favorable outcomes. This case underscores the importance of considering rare entities like vulval leiomyoma in the differential diagnosis of vulvar masses to ensure appropriate management and optimal patient care.
